# Development, feeding, and sex shape the relative quantity of the nutritional obligatory symbiont *Wolbachia* in bed bugs

**DOI:** 10.3389/fmicb.2024.1386458

**Published:** 2024-05-07

**Authors:** Marius Poulain, Elodie Rosinski, Hélène Henri, Séverine Balmand, Marie-Laure Delignette-Muller, Abdelaziz Heddi, Romain Lasseur, Fabrice Vavre, Anna Zaidman-Rémy, Natacha Kremer

**Affiliations:** ^1^Université Lyon 1, CNRS, VetAgroSup, Laboratoire de Biométrie et Biologie Evolutive, UMR 5558, Villeurbanne, France; ^2^INSA Lyon, INRAE, BF2I, UMR203, Villeurbanne, France; ^3^Izinovation SAS, Lyon, France; ^4^Institut Universitaire de France, Paris, France

**Keywords:** Cimex lectularius, Wolbachia, nutritional symbiosis, host-symbiont interaction, bacterial growth dynamics, development, bacteriome

## Abstract

The common bed bug, *Cimex lectularius*, is a hemipteran insect that feeds only on blood, and whose bites cause public health issues. Due to globalization and resistance to insecticides, this pest has undergone a significant and global resurgence in recent decades. Blood is an unbalanced diet, lacking notably sufficient B vitamins. Like all strict hematophagous arthropods, bed bugs host a nutritional symbiont supplying B vitamins. In *C. lectularius*, this nutritional symbiont is the intracellular bacterium *Wolbachia* (*w*Cle). It is located in specific symbiotic organs, the bacteriomes, as well as in ovaries. Experimental depletion of *w*Cle has been shown to result in longer nymphal development and lower fecundity. These phenotypes were rescued by B vitamin supplementation. Understanding the interaction between *w*Cle and the bed bug may help to develop new pest control methods targeting the disruption of this symbiotic interaction. The objective of this work was thus to quantify accurately the density of *w*Cle over the life cycle of the host and to describe potential associated morphological changes in the bacteriome. We also sought to determine the impact of sex, feeding status, and aging on the bacterial population dynamics. We showed that the relative quantity of *w*Cle continuously increases during bed bug development, while the relative size of the bacteriome remains stable. We also showed that adult females harbor more *w*Cle than males and that *w*Cle relative quantity decreases slightly in adults with age, except in weekly-fed males. These results are discussed in the context of bed bug ecology and will help to define critical points of the symbiotic interaction during the bed bug life cycle.

## 1 Introduction

Bed bugs have undergone a major and worldwide resurgence in the number of infestations during the last decades (Doggett and Lee, 2023) due to globalization (Štefka et al., 2022), urbanization (Hwang et al., 2005), and selection of insecticide resistance (Dang et al., 2017). Bed bugs are mainly found in habitats associated with humans (Parola and Izri, 2020): individual or collective housing, habitats linked to tourism (e.g., hotels, seasonal rentals) or health (e.g., hospitals, retirement homes), but also places associated with culture (e.g., movie theaters) or transportation (e.g., trains). Bites lead to itchy lesions and rashes, and infestations can cause sleep disturbances, psychological distress, and stigma (Doggett et al., 2012). Thus, bed bugs are a growing health and socio-economic burden requiring new, alternative methods to insecticides to control their populations.

The common bed bug, *Cimex lectularius,* is a strict hematophagous pest insect. This hemimetabolous insect undergoes five nymphal stages before the final molt to adulthood. Each instar requires a blood meal to proceed to the molting in 1 week, and both sexes bite at the adult stage. Like other strict hematophagous insects or species living on a nutritionally unbalanced diet (Janson et al., 2008; Rio et al., 2017; Duron and Gottlieb, 2020), bed bugs have evolved a long-term association with endosymbionts providing essentials nutrients that they lack in their diet, notably sufficient B vitamins. As far as known, *C. lectularius* microbiota is composed of one to three endosymbionts, one of them being an obligate nutritional symbiont. The obligate endosymbiont is *Wolbachia* (*w*Cle) (Sakamoto and Rasgon, 2006) and provides two essential B vitamins (i.e., biotin and riboflavin) (Hosokawa et al., 2010; Nikoh et al., 2014; Hickin et al., 2022). The experimental depletion of *w*Cle results in a decrease in the number of laid eggs, a decrease in hatching rate, an increase in nymphal development, and a smaller adult size (Chang, 1974; Hosokawa et al., 2010; Hickin et al., 2022). The two facultative endosymbionts are a γ-proteobacterial endosymbiont (Hypša and Aksoy, 1997; Hosokawa et al., 2010) of the Symbiopectobacterium family, known as *BEV*-like, for “Bacterial symbiont of *Euscelidius* var*iegatus*” (Degnan et al., 2011; Nadal-Jimenez et al., 2022) and a torix *Rickettsia* (Potts et al., 2020; Thongprem et al., 2020; Pilgrim et al., 2021). Little is known about these facultative endosymbionts, but their effect on fitness appears limited (Hosokawa et al., 2010; Thongprem et al., 2020). All three endosymbionts are present in the ovaries, which ensure their vertical transmission through eggs (Hosokawa et al., 2010; Thongprem et al., 2020). Endosymbionts are also observed in specialized host cells called bacteriocytes. These cells form oblong-shaped organs, the bacteriomes, which are localized on the left and right side of the insect, between the fourth and fifth abdominal tergites (Hertig and Wolbach, 1924; Chang and Musgrave, 1973; Hosokawa et al., 2010; Thongprem et al., 2020).

Disrupting the nutritional symbiosis with the obligate endosymbiont *w*Cle could therefore be a promising control method, for instance by inducing a break-down in symbiosis or a stop in metabolic supplementation. To develop such a control strategy, it is essential to understand the dynamics of *w*Cle throughout the insect development, and as a function of sex and feeding. Previous studies have reported the presence of *w*Cle in the eggs, the first instars, and in the adults of both sexes (Hosokawa et al., 2010; Thongprem et al., 2020). *w*Cle relative quantity was shown to be higher in the fifth instar stage than in the first nymphal stage and to decrease in adult females starved for at least 21 days (Fisher et al., 2018). These results raise the question of the impact of development and natural aging (independent of starvation), but also of the dynamics of *w*Cle in males. Here, we thus present a detailed characterization of the interaction between *C. lectularius* and its nutritional obligatory endosymbiont *w*Cle using cohorts. We describe the dynamics of *w*Cle load in *C. lectularius*, and the effect of feeding, at all nymphal stages. We also document the development of the bacteriome during nymphal development. Finally, we quantify the evolution of the relative quantity of *w*Cle after adult emergence and specifically test the impacts of sex, aging, and starvation on the relative quantity of *w*Cle in adults.

## 2 Methods

### 2.1 Insects, feeding and rearing

The “F4” strain of *C. lectularius* was sampled in London (UK) in 2008 (Thongprem et al., 2020). Isofemale lines were created immediately after sampling and were reared on human blood in Oliver Otti’s lab (Dresden, Germany) until 2021. For our experiments, we used three of these isofemale lines, provided by O. Otti: F4-V1, F4-V6, and F4-V48. These lines were confirmed by PCR to be negative for torix *Rickettsia* and are positive for *BEV*-like. We fed bed bugs on the Hemotek^®^ system with human blood provided by the *Etablissement Français du Sang* [blood group: O or A with 17 IU.mL Sodium Heparin (BD Vacutainer); temperature: 36.5 ± 0.5°C; membrane: parafilm^®^]. Colonies were maintained in round plastic jars containing corrugated cardboard harborage shelters (41 mm × 30 mm), at 24°C, 60% relative humidity, and with a photoperiod of 12 L:12D.

### 2.2 Cohort generation and sampling for bacterial dynamics in nymphs

Couples from either F4-V1, F4-V6, and F4-V48 lines were fed weekly on 1 mL of human blood for 30 min. The cohorts were generated as follows: fed couples were isolated in 24-well plates to mate and oviposit for 5 days, then males were removed. After 2 weeks, females were removed, and nymphs were gathered in round plastic jars containing corrugated cardboard harborage shelters. For each cohort and each nymphal stage, unfed (UF) nymphs (1 to 3 nymphs per cohort) were sampled from the first nymphs hatched (#1 in Supplementary Figure S1) and the remaining nymphs were fed. Nymphs that did not feed were discarded. Sampling was completed at each nymphal stage by collecting nymphs one-day post-feeding (1DPF, #2 in Supplementary Figure S1), and five-days post-feeding (5DPF, #3 in Supplementary Figure S1). Molting to the next instar occurred 1 week after the last meal; nymphs that did not molt were discarded, and samplings were performed (UF, 1DPF, 5DPF) as described previously for the following stages (second, third, fourth, and fifth instar). Bed bugs were frozen immediately after collection and kept at −20°C until DNA extraction. A total of 168 nymphs were sampled, originating from 6 cohorts (1 F4-V1, 2 F4-V6, and 3 F4-V48).

### 2.3 Cohort generation and sampling for bacterial dynamics in adults

Nymphs at the fifth instar were taken from either F4-V1, F4-V6, and F4-V48 lines and were fed on 1 mL of human blood for 30 min. To quantify *Wolbachia* density over the nymph-to-adult transition, 5DPF fifth instars were sampled for both sexes, the sex being determined at that stage by a combination of approaches described previously in Langer et al. (2020). One week after the fifth instar feeding, residual nymphs were discarded.

To determine the effect of sex, feeding, and aging on *Wolbachia* density, 1DPF male and female adults were sampled, and populations were divided equally into two round plastic jars. One jar was fed weekly (condition: weekly feeding), while the other one was not fed (condition: starvation after feeding on day 1). Each week, for 4 weeks, males and females of both jars were sampled at the date corresponding to 1DPF for the fed population (Supplementary Figure S2). Bed bugs were frozen immediately after collection and kept at −20°C until DNA extraction. A total of 146 fifth instars and adults were sampled, originating from 6 cohorts (1 F4-V1, 2 F4-V6, and 3 F4-V48).

### 2.4 DNA extraction

DNA was extracted using the MACHEREY-NAGEL NucleoSpin^®^ 96 Tissue kit, following the protocol for genomic DNA from tissue, except for small individuals (i.e., from first to third instars). Small instar samples were placed in 0.2-mL tubes with three 1.5-mm stainless steel beads while large instar samples were placed in 2-mL tubes with one 5-mm diameter bead. All samples were left for 15 min at −80°C before being ground with a Tissue Lyzer (Retsch, Qiagen) for 2 min at 20 Hz. The following steps of the protocol followed the supplier’s recommendations, except for the small individuals. Indeed, for small individuals, the volume of lysis and wash buffers was divided by 3 compared to the recommendations and compared to the samples considered as large (i.e., from the fourth nymphal stage to the adult stage). For all samples, pre-lysis was carried out for 2 h at 56°C, washes were performed under vacuum, while elution was performed by centrifugation at 5600 g for 2 min with 100 μL of elution buffer. Eluted DNA was stored at −20°C until qPCR quantification.

### 2.5 Detection of symbionts using PCR amplification

Conventional PCR was used to verify the presence/absence of symbionts. The *w*Cle 16S rRNA gene [INT2F, INT2R; 136 bp (Sakamoto and Rasgon, 2006)], the *γ-proteobacteria* 16S rRNA gene [BEV_F, BEV_R; 420 bp (Hosokawa et al., 2010)], the torix *Rickettsia gltA* gene [RiGltA405_F, RiGltA1193_R; 786 bp (Pilgrim et al., 2017)], and the *C. lectularius* ribosomal protein (*RPL18*) gene [RPL18F, RPL18R; 137 bp (Fisher et al., 2018)] were PCR-amplified as follows on a subset of unfed fifth instars (*n* = 10) and on one control sample known to be infected by the three symbionts. A 25-μL reaction containing 2.5 μL 10X DreamTaq^®^ Green Buffer, 0.5 μL of dNTP (10 μM), 0.1 μL of DreamTaq^®^ DNA Polymerase (5 U/μL), 0.5 μL of each primer set (10 μM) (primer sequences in Supplementary Table S1), and 2 μL of template DNA (previously diluted to 1/20) was prepared. All PCR reactions were performed in a Bio-Rad C1000 Touch™ thermal cycler with the following program: an initial denaturation step at 95°C for 5 min, followed by 35 cycles of 95°C for 30 s, Tm °C for 30 s and 72°C for 1 min. A final extension step of 72°C for 5 min was included. To visualize the amplicons, 5 μL of the PCR products were electrophoresed at 100 mV for 25 min (TBE 0.5X, 1% agarose, 0.5% BIOTIUM GelRed^®^ Nucleic Acid Gel Stain).

### 2.6 *Wolbachia* quantification using real-time PCR

A real-time quantitative PCR (qPCR) duplex assay targeting the *w*Cle-*16S* rDNA and the bed bug *RPL18* gene was used to obtain relative quantification of *w*Cle in each bed bug. The 10-μL reaction mix contained 5 μL SsoAdvanced^™^ Universal Probe Supermix (Bio-Rad), 500 nM of each forward and reverse primer, 300 nM of each probe, and 2 μL of extracted DNA previously diluted to 1/20. We used primers and probes previously described (Supplementary Table S1). The probes were provided by IDT DNA Technologies and included two quenchers, Iowa Black^®^ at the 3′ end, and another internal quencher called ZEN^™^. Each probe also had a specific fluorophore at the 5’ end, FAM for *w*Cle, and HEX for the *RPL18* gene of *C. lectularius*. Real-time qPCR was performed in QuantStudio 6 Flex^™^ (Applied Biosystems) with the following program: an initial denaturation step at 95°C for 30 s, followed by 40 cycles of 95°C for 10 s, 60°C for 30 s and 72°C for 30 s. The qPCR assay efficiency (primer efficiency in Supplementary Table S1) was determined and confirmed in every run by constructing a standard curve using serial dilutions of a purified and quantified amplicon of each target. In addition, the specificity and absence of inhibitors in the samples were assessed upstream using dilutions of DNA extracts produced under the same conditions.

### 2.7 Statistical analysis of the relative quantity of *Wolbachia*

Each qPCR measurement was made in duplicate. Values for which the delta of quantification cycles (Cq) between replicates was greater than 0.5 cycles or the values of Cq were higher than 35 cycles were discarded [*w*Cle-16S: 7.14% discarded (*n* = 12), RPL18: 5.36% discarded (*n* = 9; 8 in common with *w*Cle-16S)]. Most of these discarded samples were young nymphal stages (71.42% of discarded are first instars).

The relative quantity (RQ) of *Wolbachia* within the insect was calculated based on equation one of Pfaffl (2001) where the ratio between the quantity of insect gene (reference) and *w*Cle gene (target) relied on the PCR efficiency (E) and the number of Cq:


$$RQ=\frac{E\mathrm{refe}rence^{\mathrm{Cqreference}}}{\mathrm{Etarge}t^{\mathrm{Cqtarget}}}$$

We used the R software (version 4.1.0) for all analyses (R Core Team, 2024) and the ggplot2 R package (version 3.4.4) for all plots (Wickham, 2016). To test the effect of different factors (i.e., development, feeding, sex) and their interactions on relative *w*Cle quantities, we analyzed log_10_-transformed data using a linear mixed effect model with the lme4 R package (Bates et al., 2015).

For nymphal (N) samples, we tested the following model: lmer [log_10_(RQ) ~ Age_N + Feeding_State + Age_N:Feeding_State + (1|Cohort)], where Age_N is a quantitative variable associated to nymphal development (weeks), Feeding_State is a qualitative factor associated to blood feeding status (UF/1DPF/5DPF), and Cohort is a random factor linked to replicate sampling (A to F). Unfed (UF) first instars are considered as references in the model.

For adult (A) samples, because the feeding factor varies only after the adult’s first blood meal, we first tested on fifth instars 5DPF and adults 7DPF of both sexes (*n* = 26) the effect of development (fifth instar to adult) on the relative quantity in *w*Cle in both sexes, with the following model: lmer [log_10_(RQ) ~ Age + Sex + Sex:Age + (1|Cohort)], using the fifth instar females as reference. We then tested on adults only the effect of aging, blood feeding, and sex, with the following model: lmer [log_10_(RQ) ~ Age_A + Sex + Feeding:Age_A + Sex:Age_A + Feeding:Sex:Age_A + (1|Cohort)], using the weekly fed-first week females as reference. Age and Age_A are quantitative variables associated with development and aging, respectively; Sex is a qualitative factor (female, male); Feeding is a qualitative factor associated to blood feeding status (fed, unfed), whose dynamics depends on the aging/duration of starvation; and Cohort is a random factor linked to replicate sampling (G to L).

Normality and homoscedasticity were checked graphically on residuals for each fitted model. Residuals were also checked for homogeneity of variance. Statistics for global effects of factors and their interactions are reported in Supplementary Tables S2, S4, S5. Model coefficients are reported with their mean ± confidence intervals (0.95). Interaction effects can be interpreted as an additive effect compared with the reference. Because calculating *p*-values from linear mixed effect model is complex and subject to controversy (Wasserstein and Lazar, 2016; Wasserstein et al., 2019), p-values estimated using the lmerTest R package (Kuznetsova et al., 2017) are only specified in Supplementary Tables S2, S4, S5.

### 2.8 *Wolbachia* localization using Fluorescence *In situ* Hybridization (FISH)

We documented the localization of *w*Cle for each nymphal stage after molting (10 nymphs per instar) in the F4-V48 line, using the FISH technique (Thongprem et al., 2020). Tissues were dissected in 1X PBS and preserved immediately in Carnoy solution (chloroform: ethanol: glacial acetic acid = 6:3:1) overnight. All samples were cleared by incubation in 6% H_2_O_2_ in ethanol until the body was transparent, i.e., for at least 24 h for first and second instars, and up to 5 weeks for third to fifth instars. We then used a tungsten micro-needle to make micropores in the nymph cuticle to allow the fluorescence probes to diffuse through the cuticle during the hybridization step. The samples were hybridized by incubating the tissues overnight in a hybridization buffer (20 mM Tris–HCl pH 8.0, 0.9 M NaCl, 0.01% Sodium dodecyl sulfate 30% formamide) with 10 pmol/mL of rRNA specific probes for *w*Cle (Hosokawa et al., 2010; probe sequences in Supplementary Table S1). After incubation, tissues were washed in buffer (0.3 M NaCl, 0.03 M sodium citrate, 0.01% sodium dodecyl sulfate) and mounted onto a slide using fluoro Gel with DABCO (Electron Microscopy Science) as a mounting medium. Slides were then observed under Leica THUNDER Imager 3D Assay epifluorescence microscope.

### 2.9 Evolution of bacteriome size over development

Length of bacteriome and thorax were measured on ImageJ 1.53c (Schneider et al., 2012) taking the maximum length (L_b_) and width (W_b_) of both bacteriomes, and the length (L_p_) and width (W_p_) at the middle of the pronotum. Relative bacteriome sizes were calculated by the ratio between pronotum estimated area (L_p_ ×W_p_) and the mean estimated area (π × (1/2) L_b_ × (1/2) W_b_) of both bacteriomes. We used the R software (version 4.1.0) for all analyses and plots (R Core Team, 2024).

To test the effect of age on absolute area of the bacteriome, we analyzed data using a Linear Model (LM). We tested the following model: lm (log_10_(Mean_area_bact) ~ Stage), where Stage is a qualitative factor associated to nymphal development (first to fifth instar) and Mean_area_bact is the mean of the estimated area of both bacteriomes in a nymph.

To test the effect of the age on the relative area of the bacteriome, we analyzed data using the following model: lm (Mean_ratio_area ~ Stage), where Mean_ratio_area is the mean of the estimated area of both bacteriomes relatively to the estimated area of the thorax in the nymph.

Normality and homoscedasticity were checked graphically on residuals for each fitted model. Residuals were checked for homogeneity of variance. LM statistics were given for global effects of factors. To compare the means of either the area or the relative size of the bacteriome between stages, we performed post-hoc Tukey tests (see Supplementary Table S3 for the complete output of each model).

### 2.10 Characterization of cryofixed bacteriomes using electro-microscopy (EM)

We performed the following manipulations at the *Centre Technologique des Microstructures* (CTμ, Université Claude Bernard Lyon 1, France). To avoid losing the samples during the procedure, bacteriomes of fifth instar females were dissected in PBS together with the proximal ovary and the surrounding cuticula. Samples were placed into specimen carriers previously covered with 0.5% lecithin in chloroform. Carriers were directly loaded into the HPM100 high pressure freezer and fast frozen. After freezing, samples were placed into a Leica EM AFS II Freeze substitution machine and incubated at -90°C for 30 h in substitution buffer (1% osmium tetroxide, 0.25% uranyl acetate, 0.5% glutaraldehyde, 1.5% H_2_O in acetone) and then gradually heated to -30°C (+5°C/h) and maintained at that temperature for 24 h. Samples were washed with -30°C cold acetone and then gradually heated to 20°C (+10°C/h). Samples were placed in 25% Epon^™^ (Epoxy substitute embedding medium kit from Sigma-Aldrich®) in acetone for 3 h, 50% Epon^™^ in acetone for 17 h, 75% Epon^™^ in acetone for 3 h, then in three different baths of pure Epon^™^ for a total of 24 h. Infiltration and embedding in 1.7% benzyl dimethyl amine in Epon™ resin were performed during 4 days at 60°C.

Sectioning was performed on Leica EM UC7 ultramicrotome using a diamond knife (Diatome) and mounted on uncoated copper grids. Ultra-thin sections (70 nm) for Transmission Electro-microscopy (TEM) were observed at 120 keV using a JEOL 1400 electron microscope. Electron micrographs of standard sections were taken with GATAN DigitalMicrograph software (Pleasanton, CA) and further analyzed using ImageJ 1.53c.

## 3 Results

### 3.1 The relative quantity of *w*Cle increases exponentially over nymphal development

To analyze the dynamics of the obligatory endosymbiont *w*Cle during nymphal development, we measured by qPCR duplex assay the relative quantity of *w*Cle in 157 nymphs at different stages of development. *w*Cle quantity was below the level of detection in 2.55% of the samples (*n* = 4), which were all first instars. We tested the effect of development and feeding status on *w*Cle relative quantity on the remaining 153 samples, considering the variability observed between cohorts as a random effect in the model (Supplementary Figure S3).

**Figure 1:**
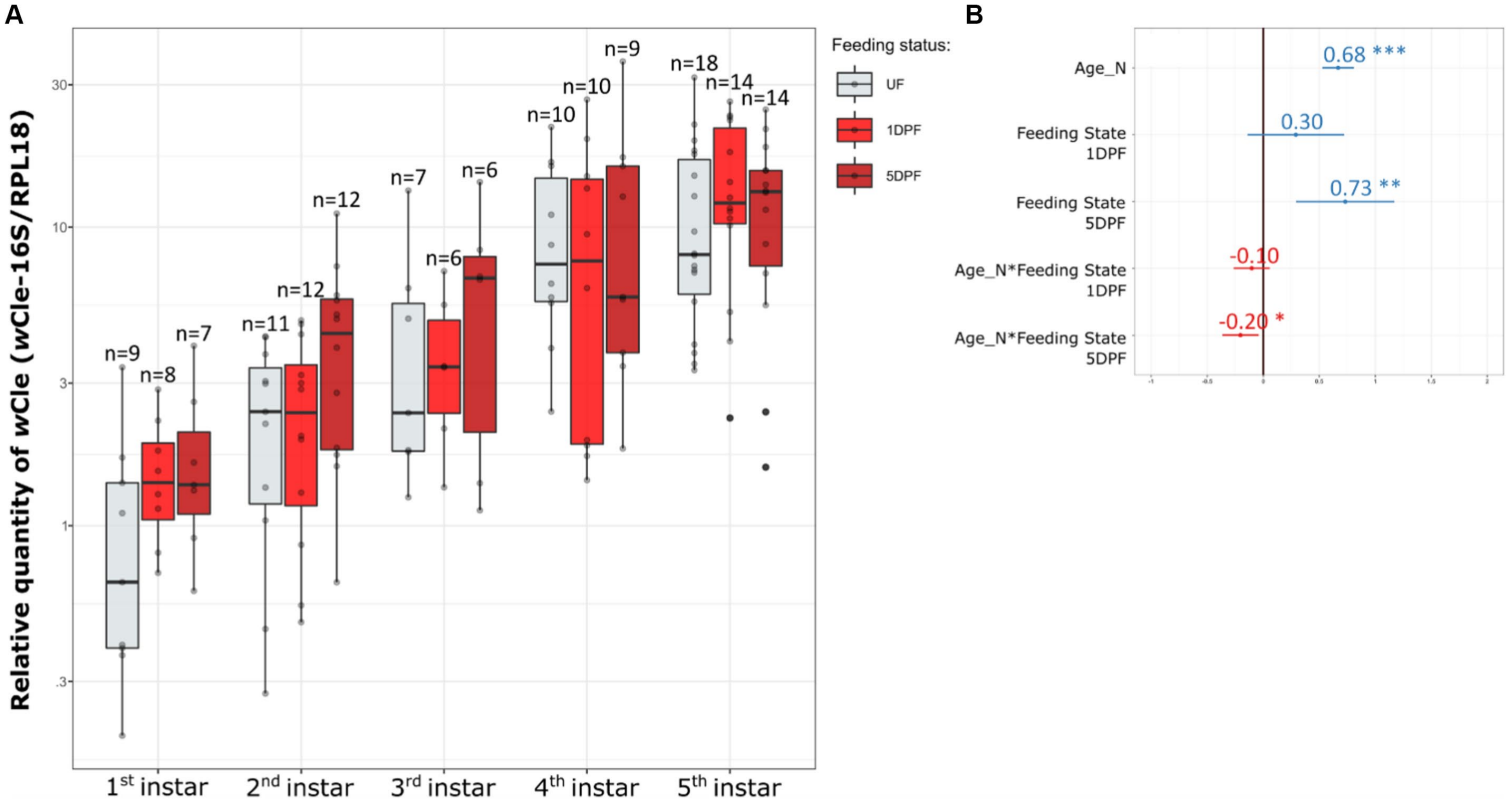
Dynamics of the *w*Cle relative quantity in nymphal bed bugs over development and associated statistics. **(A)** *w*Cle relative quantities of unfed (UF) newly molted nymphs, 1-day post-feeding nymphs (1DPF), and 5-day post-feeding nymphs (5DPF) are represented as boxplots for each nymphal instar. Each boxplot represents n = 6–18 individual nymphs (1 to 3 sampled nymphs per cohort). **(B)** Resume statistics of the mixed model log_10_(RQ) ~ Age_N + Feeding_State + Age_N:Feeding_State + (1|Cohort), where Age_N is a quantitative factor (number of weeks) and Feeding_State a qualitative factor (UF, 1DPF, 5DPF, with UF set as reference). Estimate values and their associated confidence intervals are indicated on the panel (blue when positive effect, red when negative effect). As example for this analysis: log_10_(RQ) increases by 0.68 ± 0.11 each week for UF individuals, log_10_(RQ) increases by 0.73 ± 0.45 between the UF reference and 5DPF, and log_10_(RQ) increases by 0.68–0.20 = 0.48 each week for 5DPF individuals. For detailed statistics, see Supplementary Table S2.

The log_10_-transformed relative quantity of *w*Cle linearly increased with age (Figure 1) with a slope equal to 0.68 ± 0.11 (confidence interval at 0.95), equivalent to an increase by 4.68 of the relative quantity of *w*Cle at each nymphal stage; the log_10_-transformed absolute quantity of *w*Cle, as measured by quantification of its *16S* rDNA, increased ~2 times more strongly than that of the bed bug gene *RPL18* genes (Supplementary Figure S4). This result confirms an increase in the *w*Cle quantity relative to the number of host cells over nymphal development.

The blood feeding status also impacted the relative *w*Cle quantity, with an increase of 0.73 ± 0.45 on log_10_-transformed relative quantity (i.e., equivalent to an increase by 5.37 of the relative quantity) between unfed and 5DPF (Days Post Feeding) nymphs. This positive impact of blood feeding on the *w*Cle relative quantity dimmed as the nymph was growing (see interaction Age_N*Feeding State 5DPF Figure 1B).

### 3.2 The relative size of the bacteriome remains stable during nymphal development

**Figure 2:**
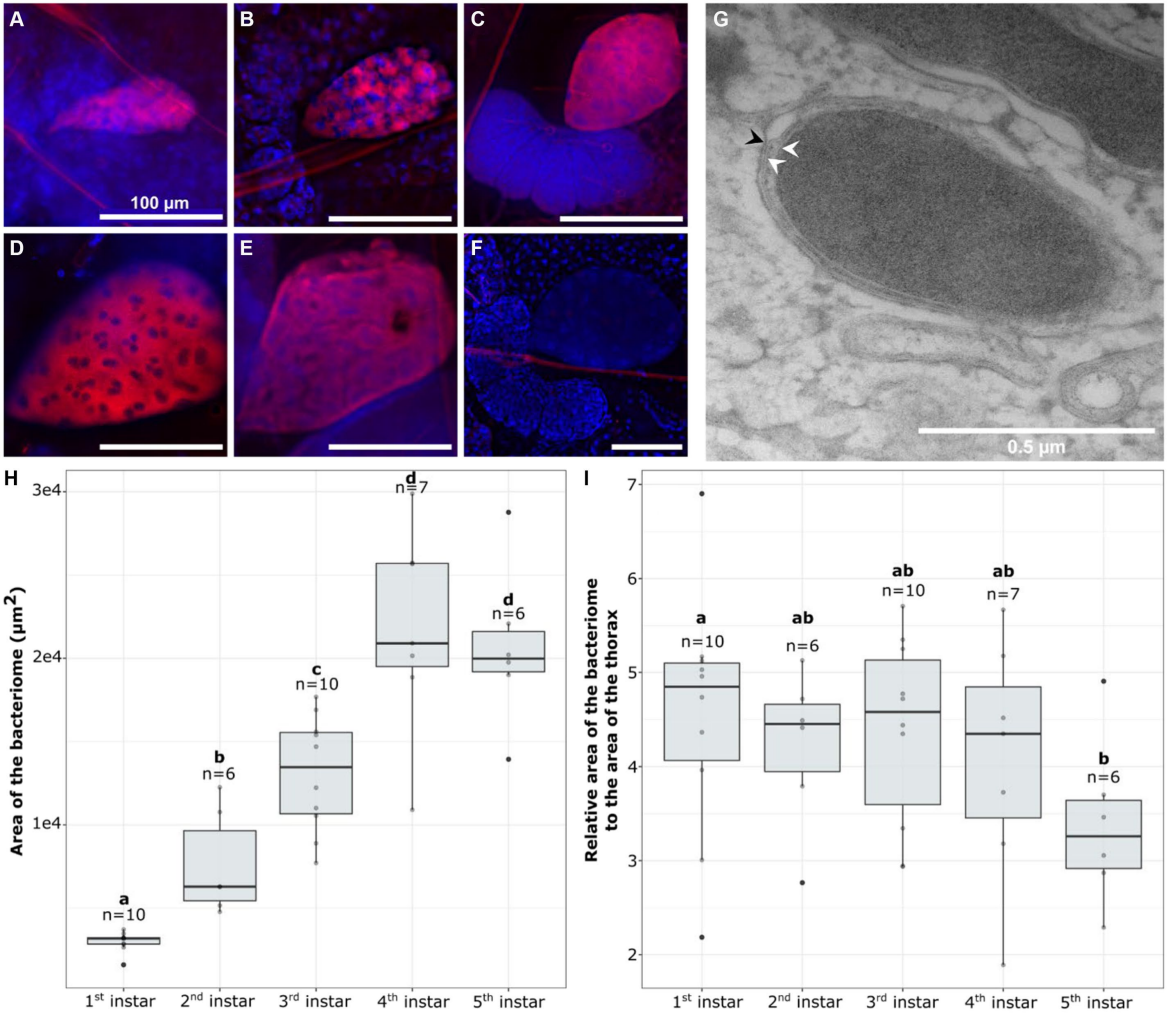
Visualization of the bacteriome and endosymbiont dynamics over bed bug development. **(A–F)** FISH visualization of *w*Cle in bacteriomes **(A)** first instar; **(B)** second instar; **(C)** third instar; **(D)** fourth instar; **(E)** fifth instar; **(F)** Negative control (without probe) of a third instar bacteriome. In red: *w*Cle; in blue: bacteriocyte nuclei (DAPI). Scale bars: 100 μm. **(G)** TEM of *w*Cle in a fifth instar female. White arrows show the two inner membranes of the endosymbiont, while the black one shows the peripheric third vacuolar membrane. **(H,I)** Dynamics of the bacterial size over development: **(H)** absolute area or **(I)** relative area of the bacteriome at each nymphal instar (first to fifth). Each boxplot represents *n* = 6–10 individual nymphs (F4-V48 line). Boxplots sharing the same letter are not significantly different (*t*-test, *p* > 0.05). For statistics, see Supplementary Table S3.

To determine if the increase in *w*Cle relative quantity is associated with a change in the bacteriome shape or an over-growth of endosymbiont within the bacteriome, we performed Fluorescence *In situ* Hybridization (FISH) using probes targeting the endosymbiont 16S rRNA. *w*Cle was detected in bacteriomes of each sex at all instars during nymphal development (Figures 2A–F). All the bacteriocytes observed within a bacteriome were infected, and their cytoplasm was packed with *w*Cle (Figures 2A–E; Supplementary Figure S5). TEM observation performed on fifth instar females confirmed that *w*Cle exhibits a three-layer membrane presumably composed of the two membranes of the symbiont and an individual vacuolar membrane of insect origin similar to what has been described 50 years ago (Chang and Musgrave, 1973) (Figure 2G).

To determine if the increase in *w*Cle relative quantity is associated with an increase in symbiotic organ size, we measured the size of the bacteriome in 39 nymphs (*n* = 6–10/stage). While the bacteriome absolute area doubled at each stage between the first and fourth instars (Figure 2H), its relative area to the thorax remained unchanged between the different developmental stages (Figure 2I).

### 3.3 The dynamics of *w*Cle is positively impacted by feeding in males but not in females

**Figure 3:**
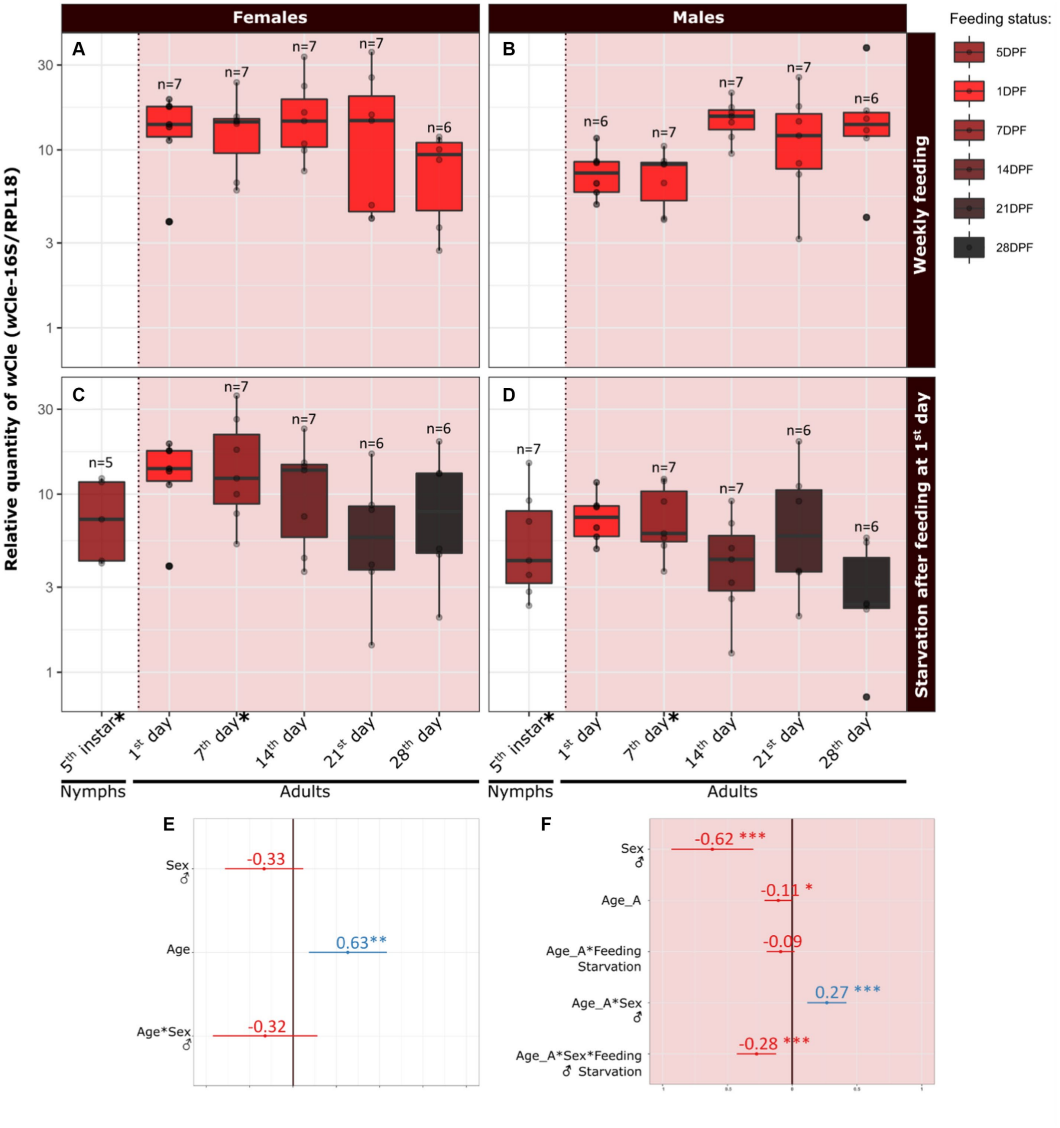
Dynamics of the relative quantity of *w*Cle from the fifth instar to adulthood, and during 5 weeks of adulthood. Concerning the relative quantity dynamics during last metamorphosis (i.e., between fifth instar (5DPF) and adulthood (7DPF) according to sex), the dataset used is marked with an asterisk on panels C and D. This first dataset was analyzed using a linear mixed model: log_10_(RQ) ~ Age + Sex + Sex:Age + (1|Cohort), where Age is a quantitative factor (weeks) and Sex a qualitative factor (female, male; female set as reference). Resume statistics are indicated on panel **(E)** Concerning the relative quantity dynamics in adult bed bugs according to sex, aging and feeding, the dataset used is presented in the red squares on the right of the dashed lines: relative quantity of *w*Cle in **(A)** females and **(B)** males fed every week (collection: 1DPF), or in **(C)** females and **(D)** males starved after the first-week meal (collected concomitantly with fed individuals). This latter dataset was analyzed using a linear mixed model log_10_(RQ) ~ Age_A + Sex + Feeding:Age_A + Sex:Age_A + Feeding:Sex:Age_A + (1|Cohort), where Age_A is a quantitative factor (weeks), Sex a qualitative factor (female set as reference) and Feeding is a qualitative factor (weekly fed, starved; weekly fed set as reference). Resume statistics are indicated on panel **(F)**. Each boxplot represents *n* = 6–7 individuals. Estimate values and their associated confidence intervals are indicated on panels E and F, respectively for each statistical analysis (blue when positive effect, red when negative effect). For detailed statistics, see Supplementary Tables S4, S5.

We first aimed to determine whether the increase in *Wolbachia* relative quantity during nymphal development persists after the last molt (Figures 3A–D). We thus measured the relative quantity of *w*Cle in fifth instars (5DPF) and newly emerged adults (7DPF) a few days after feeding (data used symbolized by * on Figure 3C (females) and Figure 3D (males); statistics: Figure 3E). We showed a significant increase with development, as log_10_(RQ) of adult females increased by 0.63 ± 0.42 (equivalent to an increase by 4.57 of the relative quantity) compared to their fifth instar counterparts. No significant effect of sex on bacterial relative quantity was detected in this developmental window.

We then focused on adults and determined the effect of sex, aging, and feeding on the relative quantity of *w*Cle. We chose a weekly feeding protocol to mimic the natural feeding rate of bed bugs (Reinhardt and Siva-Jothy, 2007; Saveer et al., 2021). We thus measured the relative quantity of *w*Cle weekly during 28 days after emergence, in weekly fed or starved adults of each sex (total = 119 adults) and analyzed data using a global statistical model (Figure 3F). The relative quantity of *w*Cle slightly decreased with age in females for both feeding conditions (slope on log_10_-transformed data = −0.11 equivalent to a decrease of 0.78 each week; Figures 3A,C). In males, while the relative quantity of *w*Cle also decreased with time in starved males (slope on log_10_-transformed data = 0.16–0.28 = −0.12, equivalent to a decrease of 0.76 each week; Figure 3D), it increased with time in weekly-fed males (slope on log_10_-transformed data = 0.27–0.11 = 0.16, equivalent to an increase of 1.44 each week; Figure 3B), showing complex interactions between sex, age, and feeding conditions. Overall, males had nevertheless significantly less *w*Cle than females, as log_10_(RQ) of males was 0.62 ± 0.31 times less the one of females (equivalent to a 4,2-fold difference in relative quantity, Figures 3A–D,F). The log_10_-transformed absolute quantities of *Wolbachia* showed similar patterns (Supplementary Figure S6).

## 4 Discussion

In this study, we described the dynamics of *w*Cle, a nutritional obligatory symbiont of bed bugs. We observed that: (a) the largest increase in relative *w*Cle quantity occurred during the nymphal development; (b) this increase was exponential and positively impacted by the development of nymphal instars and the feeding status; (c) the size of the bacteriome increased isometrically with the development of the nymph; (d) except for continuously fed males, *w*Cle relative quantity decreased slightly during the first 4 weeks of adulthood, independently of the feeding status.

This study shows that the increase in *w*Cle relative quantity was exponential over nymphal development. Once adulthood was reached, *w*Cle relative quantity started to slightly decrease, except in weekly-fed males. These data complete and corroborate the results obtained by Fisher et al. (2018), which showed that *w*Cle relative quantity was higher in the fifth nymphal stage than in the first nymphal stage, but did not precisely quantify the dynamic between these two instars. Their data indicated an increase of ~1.6-fold in *w*Cle relative quantity between the end of the first instar and the start of the fifth instar in Jersey City and Harold Harlan strains. The increase was stronger in our F4 strain, with a ~ 6.4-fold increase in relative quantity. In the adult stage, our data confirm the decrease they reported in starved females. However, a stronger tendency was observed in Fisher et al.’s work, as titers in three-week starved females approached the low titer observed in first instars, while in our study, the decrease in the *w*Cle titer in four-week starved females only approached the titer measured in fifth instars. Fisher et al. (2018) observed that one of their two strains retained more *w*Cle through starvation than the other. One possible explanation could be differences in the way strains respond to starvation, as we used here the F4 strain (Thongprem et al., 2020) that is different from the strains used in the work of Fisher et al. (2018).

In this study, the increase in *w*Cle relative quantity in nymphs is associated with an increase in the absolute bacteriome size, but not in its relative size. These observations suggest an increase in the number of bacteria in each bacteriome and could result from two non-exclusive mechanisms: an increase in the bacteriocyte number, or an increase in the bacteriocyte size. The respective contribution of these two alternatives is difficult to evaluate in a species such as the bed bug. Indeed, bacteriocytes form very cohesive bacteriomes and it would require dissociating the organ in order to count and measure bacteriocytes. An increase in endosymbiont relative quantity from egg to adult stage (i.e., along nymphal, larval or pupal stages, depending on the insects) has been reported in several other nutritional endosymbiotic associations, such as the tsetse fly *Glossinidia glossina*/*Wigglesworthia glossinidia* (Rio et al., 2006), the cereal weevil *Sitophilus oryzae*/*Sodalis pierantonius* (Vigneron et al., 2014), or the pea aphid *Acyrtosiphon pisum*/*Buchnera aphidicola* (Simonet et al., 2016). In the pea aphid and the weevil, this increase was associated with a change in the number of the bacteriocytes or bacteriomes. In the *A. pisum*/*B. aphidicola* association, where bacteriocytes do not group into a cohesive bacteriome (Buchner, 1965; Douglas, 1989), the increase in the endosymbiont load during development was shown to correlate with both an increase in the quantity and in the size of bacteriocytes (Simonet et al., 2016, 2018). While bacteriocyte mitotic activity has not yet been reported, bacteriocyte enlargement and polyploidy has been proposed in several models to participate to bacteriome growth (Koch, 1959; Douglas, 1989; Moran et al., 1998; Baumann et al., 2006; Orr-Weaver, 2015; Alarcón et al., 2022; Nozaki and Shigenobu, 2022). Further experiments are needed to analyze the precise cellular mechanism of bacteriome growth in the bed bug. Alternatively, the increase in bacterial relative density could be linked to an increase in bacterial load in ovaries during development. To go further, it would be interesting to perform individual bacterial quantifications in bacteriomes and ovaries/testis. Unfortunately, it is technically very difficult to sex bed bugs and dissect bacteriomes in the early nymphal stages.

We noticed that blood intake had a positive effect (after 5 DPF) on the *w*Cle relative quantity in nymphs. This could indicate that blood ingestion brings profuse nutritional elements for *w*Cle proliferation. Based on this and previous works (Hosokawa et al., 2010; Nikoh et al., 2014; Hickin et al., 2022), the following succession of critical steps in the interaction could be considered: i) blood ingestion allows the increase in bacterial load, ii) this increase allows higher provision of B vitamins by the host, iii) the host molt once a critical threshold in B vitamin is reached, one week post blood-feeding. However, measuring the dynamic of *w*Cle in week-starved nymphs will be required to demonstrate that it is indeed the blood ingestion that enhances the developmental increase in *w*Cle, and that it is not simply due to aging. In the strict hematophagous hemipteran *Rhodnius prolixus*, the blood-meal stimulates the molt through humoral factors and neuronal signals generated by stretch receptors in the gut (Adams, 1999; Lange et al., 2022). Hence, another possibility would be that blood feeding in bedbugs impacts both the insect hormonal signaling regulating the molt and the bacterial load, coordinating an increase in bacterial quantity with the molting process.

After the increase in the *w*Cle relative quantity in nymphs up to adult emergence, its decrease in both weekly fed and starved females is intriguing. The decrease observed in starved females does not reach the first instar’s quantity of *w*Cle like in Fisher et al. (2018). Because *w*Cle-synthetized B vitamins are necessary for fecundity and egg viability, we could expect either an increase or at least a steadiness in *w*Cle relative quantity in sexually mature fed females [i.e., ~5 days post emergence (Mellanby, 1939; Johnson, 1941; Davis, 1964)]. While the same pattern was observed between starved adults and weekly fed females, the slow decrease of *w*Cle could have different origins. In starved adults, nutritional scarcity could prevent the allocation of sufficient resources to endosymbiont growth or maintenance. Additionally, nutritional stress could trigger active mechanisms of endosymbiont elimination, including recycling of the symbionts for nutritive needs. For example, starved adults could engage in *w*Cle lysis by autophagy to retrieve nutrients through endosymbiont digestion during periods of nutritional scarcity. This process, known as wolbophagy, drives the elimination of the few damaged *Wolbachia* in healthy cells under stress conditions, and has been described in *Drosophila* (Deehan et al., 2021; Hargitai et al., 2022) but not in *Wolbachia*-nutritional endosymbioses. Drastic endosymbiont clearance by autophagy has been described in several associations, such as in some aphids (Hinde, 1971), the whitefly (Wang et al., 2022), or the carpenter ant (Gonçalves et al., 2020). This phenomenon is particularly exemplified in cereal weevils where the endosymbiont *S. pierantonius* grows dynamically during early adulthood, and provides essential amino acids for the host cuticle synthesis (Vigneron et al., 2014; Dell’Aglio et al., 2023). However, these gut-associated endosymbionts are eliminated through apoptotic and autophagic mechanisms, starting 1 week after adult emergence, when the cuticle is fully synthetized and functional (Vigneron et al., 2014). The moderate decrease in *w*Cle quantity we observed along 4 weeks of adulthood in the bed bug could be the result of a moderate and progressive wolbophagy, which does not exclude a more drastic elimination later in the life cycle. To note, the mechanisms of transfer of nutrients from the endosymbionts to the host, especially B vitamins, remain unidentified so far in the bed bug-*w*Cle association, and could themselves rely on moderate wolbophagy, allowing the host to maintain a *w*Cle population while “harvesting” the B vitamins from the endosymbionts accordingly to its physiological needs.

Surprisingly, the weekly fed females also present a slow decrease of *w*Cle in the 4 weeks of observation, despite having abundant nutritional resources. However, this apparent abundance of nutritional resources must be put in perspective of their physiological needs. Indeed, weekly blood-feeding increases the mating rate (Mellanby, 1939; Johnson, 1941; Saveer et al., 2021). Mating is traumatic in bed bugs (Stutt and Siva-Jothy, 2001; Reinhardt et al., 2003; Reinhardt and Siva-Jothy, 2007); a high mating rate can thus enhance their induced immunity (Siva-jothy et al., 2019) and potentially impact *w*Cle density regulation. Weekly blood-feeding also increases the number of laid eggs (Mellanby, 1939; Johnson, 1941; Saveer et al., 2021). This increased reproductive activity (mating and egg production) of weekly-fed females likely requires a higher metabolic investment that could constrain the resource allocation to the endosymbionts despite the recurrent host feeding. Finally, a part of *w*Cle could be “lost” in the vertical transmission to the eggs. Counting the number of laid eggs per female and correlating them with the remaining quantity of endosymbionts in the mother’s ovaries could be a way to confirm the potential “loss” of *w*Cle quantity by the mothers to the benefit of the transmission to progeny.

We also analyzed the *w*Cle dynamics in males, which had not been previously studied. Surprisingly, after emergence, weekly-fed males exhibited an increasing dynamics of *w*Cle relative quantity that contrasts with the decrease in *w*Cle quantities observed in starved males, but also in starved or weekly-fed females. This increasing quantity in weekly fed males is even more intriguing in males, in which the impact of an elimination of *w*Cle has a very limited effect on life history traits, such as the thorax size (Hickin et al., 2022). One hypothesis to explain the contrasting dynamics between weekly fed females and males could be that, while they both have access to food, they do likely not have similar metabolic needs, given the very costly investment the females make in reproduction. Therefore, *w*Cle could maintain and proliferate more effectively in weekly-fed males due to a higher availability of metabolic resources in these individuals.

Altogether, our main hypothesis to explain the regulation of *w*Cle behind the observed dynamics in adults would be that, in nutritional abundance conditions like weekly feeding, *w*Cle can proliferate to fulfill the host’s needs. In contrast, during critical steps of host development, when the host’s metabolic needs are higher (i.e., mating, starvation, egg formation), the symbiotic cost is in detriment of *w*Cle, whose quantity decreases. This decrease in quantity could be the result of either a decrease in proliferation due to nutritional scarcity, or an active recycling of endosymbionts by the host for example by wolbophagy. However, as described in other symbiotic associations, regulation of both *w*Cle location and load could also involve active immune mechanisms such as production of reactive oxygen species (ROS) (Pan et al., 2012; Zug and Hammerstein, 2015) or antimicrobial peptides (AMP) (Login et al., 2011) by the host.

## 5 Conclusion

This work shows a dynamics of *w*Cle that is positively impacted by blood ingestion during nymphal stages and in the adult males. This description of the dynamics of *w*Cle along the life cycle of its host allows to unveil that feeding is a critical step in the interaction, which should be considered in future research to pinpoint the best stages to decipher the molecular dialog between partners and envision the development of new symbiocides.

## Data availability statement

The raw data supporting the conclusions of this article will be made available by the authors, without undue reservation. Access to raw data and scripts: https://zenodo.org/records/10817678.

## Ethics statement

The manuscript presents research on animals that do not require ethical approval for their study.

## Author contributions

MP: Data curation, Formal analysis, Investigation, Methodology, Writing – original draft, Writing – review & editing. ER: Resources, Writing – review & editing. HH: Data curation, Methodology, Writing – review & editing, Investigation. SB: Methodology, Visualization, Writing – review & editing, Investigation. M-LD-M: Methodology, Writing – review & editing. AH: Conceptualization, Funding acquisition, Writing – review & editing. RL: Funding acquisition, Project administration, Supervision, Writing – review & editing. FV: Conceptualization, Funding acquisition, Project administration, Supervision, Validation, Writing – original draft, Writing – review & editing. AZ-R: Conceptualization, Funding acquisition, Project administration, Supervision, Validation, Writing – original draft, Writing – review & editing, Methodology. NK: Conceptualization, Formal analysis, Funding acquisition, Project administration, Supervision, Validation, Writing – original draft, Writing – review & editing, Data curation, Methodology.
