# Supplementary material for: Development, feeding, and sex shape the relative quantity of the nutritional obligatory symbiont Wolbachia in bed bugs
Source: Front Microbiol. 2024 May 7;15:1386458. doi: 10.3389/fmicb.2024.1386458 (PMC11106466; doi:10.3389/fmicb.2024.1386458)
Supplement: Supplementary file 1 [file Data_Sheet_1.docx]

**
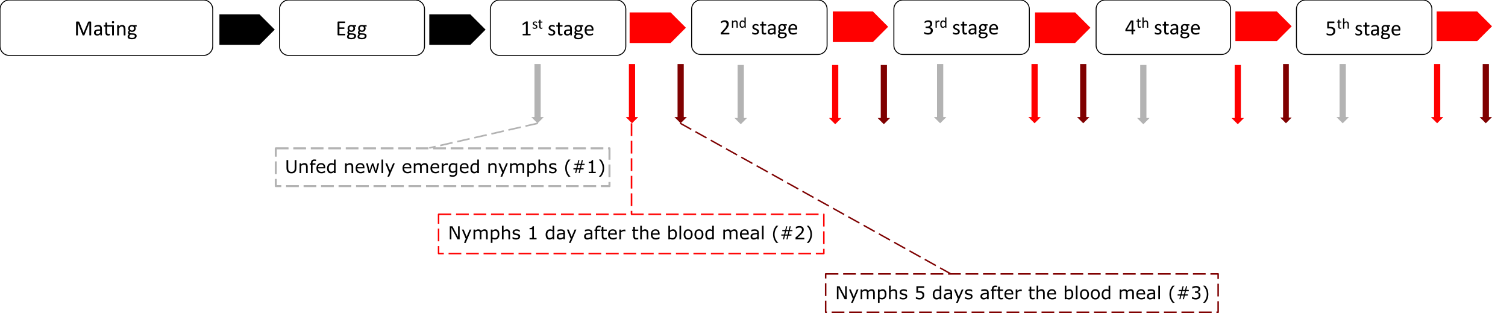
 Figure S1.** Experimental layout of the nymphal sampling. Each framed step is one weeklong. Grey arrows represent sampling of unfed newly emerged nymphs (#1: UF). Light red arrows represent sampling of nymphs one-day post-feeding (#2: 1DPF). Dark red arrows represent sampling of nymphs five-days post-feeding (#3: 5DPF).


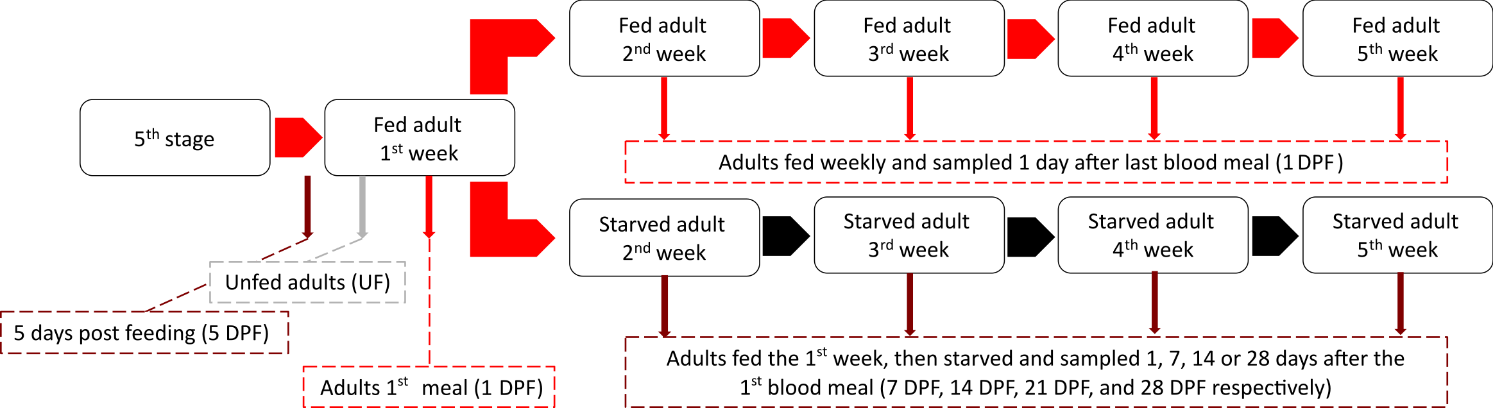
 **Figure S2.** Experimental layout of the adult sampling. Each framed step is one weeklong. Large red arrows represent a weekly feeding. Large black arrows represent the beginning of a new week without feeding. Grey arrows represent sampling of unfed newly emerged adults (UF). Light red arrows represent sampling of adults that were fed weekly and sampled 1 day after blood meal (1DPF). Dark red arrows represent sampling of fifth instars or adults that were fed 5, 7, 14, 21 or 28 days post feeding.

**
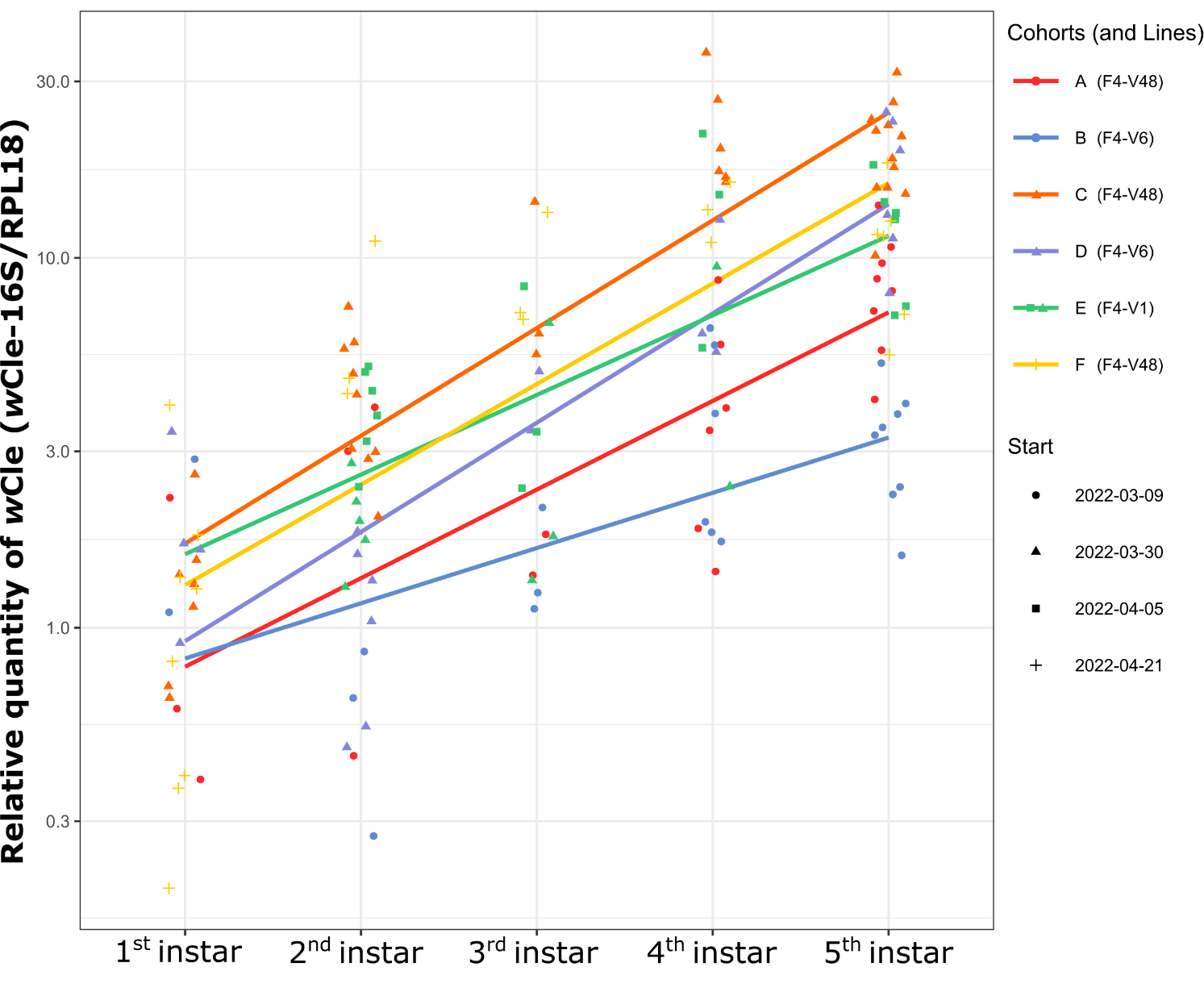
Figure S3.** Relative quantity of *w*Cle over development, according to the sampling cohort. Dots correspond to cohorts shown in the legend, and lines to linear correlations between *w*Cle relative quantity and time (i.e*.*, developmental nymphal stage).


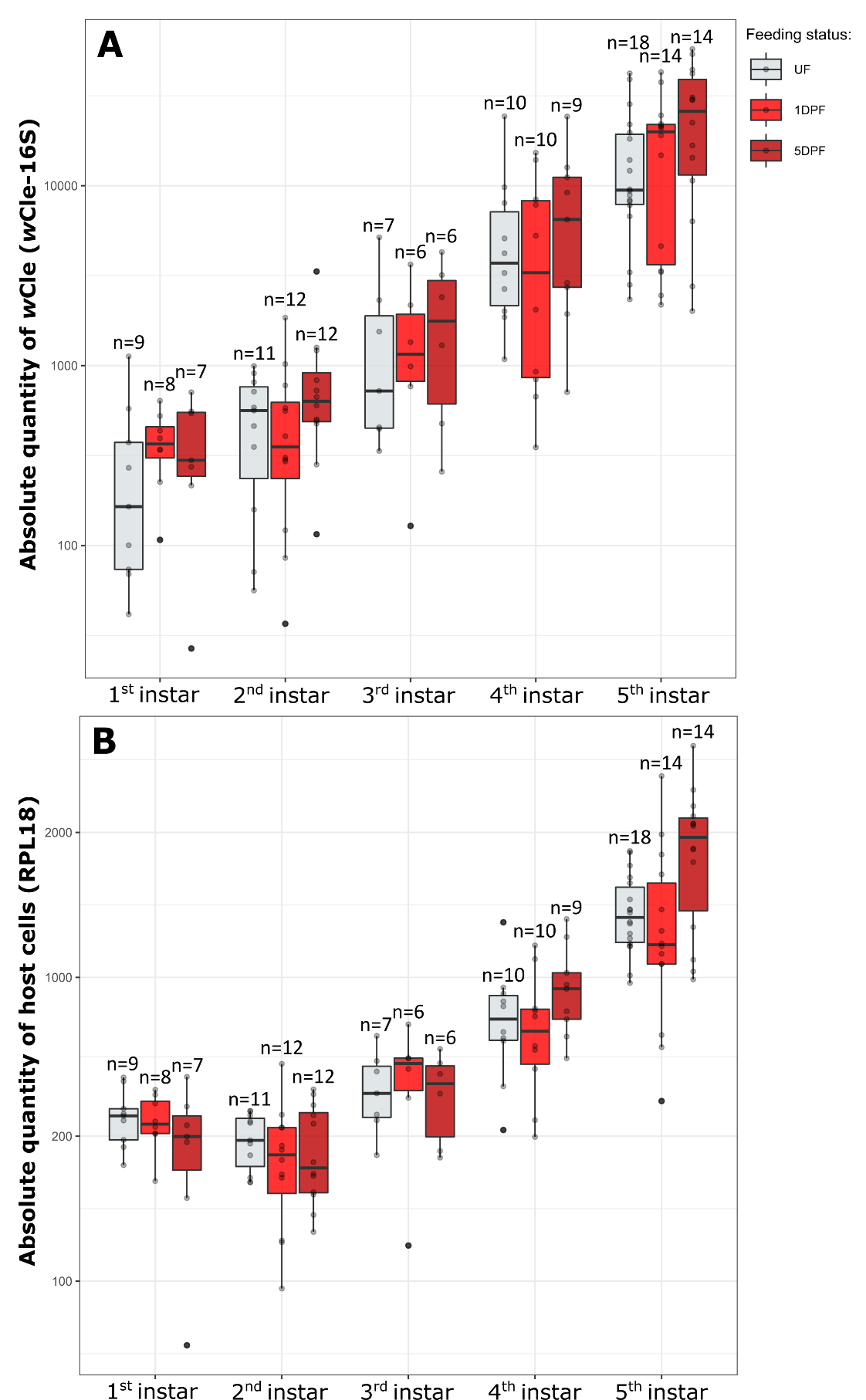


**Figure S4.** Dynamics of the absolute quantity of *Wolbachia* and host cells in nymphal bed bugs over development. Absolute quantities of **A**) *Wolbachia* *w*Cle and **B**) bed bug *RPL18* are represented as boxplots in unfed (UF) newly molted nymphs, 1-day post-feeding nymphs (1DPF), and 5-day post-feeding nymphs (5DPF). Each box represents n = 6-18 individual nymphs (1 to 3 sampled nymphs per cohort).


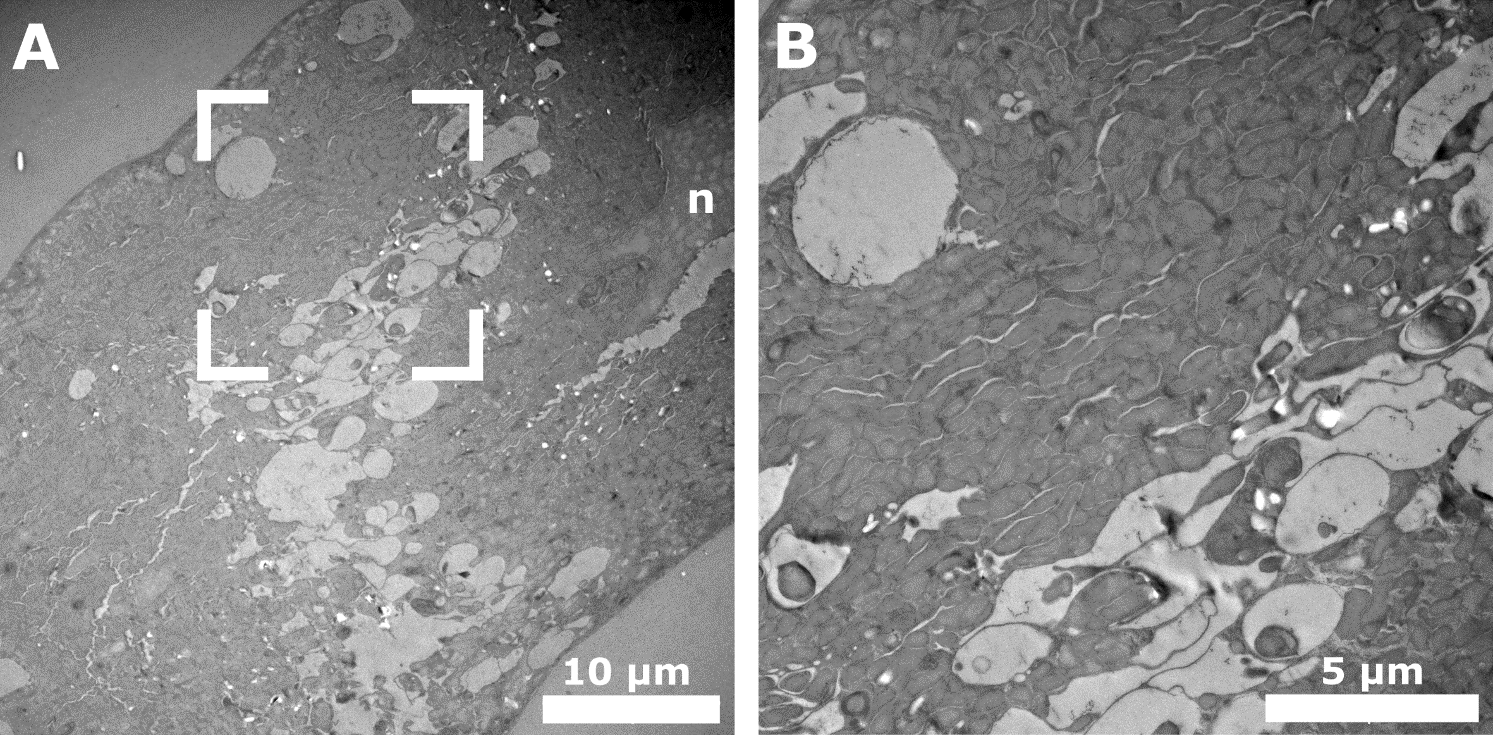
**Figure S5.** (**A**) TEM visualization of *w*Cle in a fourth instar female bacteriome. (**B**) zoom on an area of interest. *W*Cle bacteria occupy the cytoplasm and surround the nucleus (n).


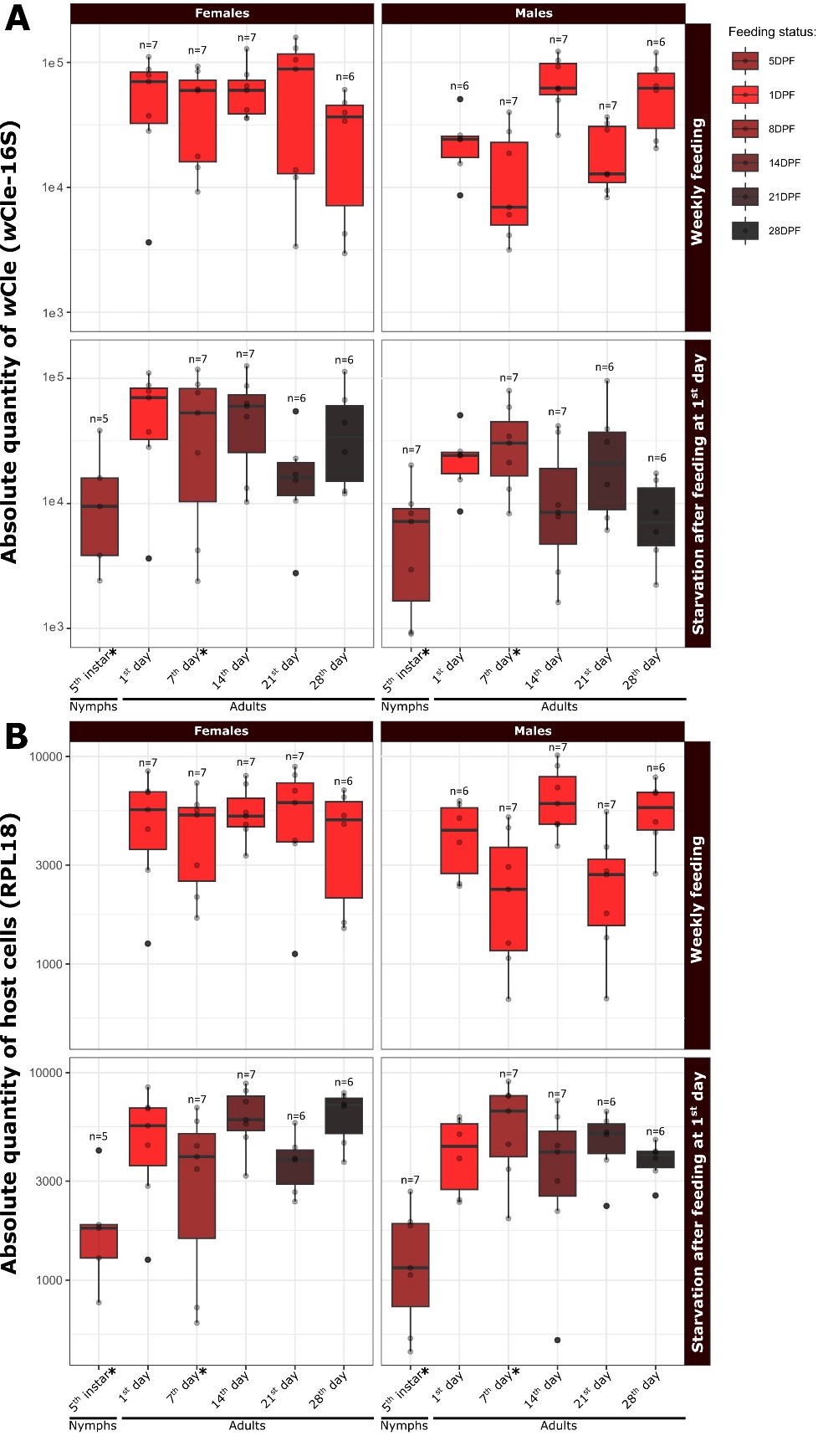


**Figure S6.** Dynamics of the absolute quantity of *Wolbachia* and host cells from the fifth instar to adulthood, and during five weeks of adulthood. Absolute quantities of **A**) *Wolbachia* *w*Cle and **B**) bed bug RPL18 are represented as boxplots for each sex and feeding conditions. Concerning the absolute quantity dynamics during last metamorphosis (i.e*.*, between fifth instar (5DPF) and adulthood (7DPF) according to sex), the dataset used is marked with an asterisk. Each boxplot represents n = 6-7 individuals.
