# Supplementary material for: Development, feeding, and sex shape the relative quantity of the nutritional obligatory symbiont Wolbachia in bed bugs
Source: Front Microbiol. 2024 May 7;15:1386458. doi: 10.3389/fmicb.2024.1386458 (PMC11106466; doi:10.3389/fmicb.2024.1386458)
Supplement: Supplementary file 2 [file Data_Sheet_2.docx]

**Table S1.** Sequences of PCR primers and fluorescence probes used in this study. All the fluorescence probes are labeled with a fluorophore at the 5’ end, indicated below in square brackets.

| Target organisms  Target gene | Primer/Probe name | Sequence (5’-3’) | qPCR Efficacy  (%) | Tm  (oC) | Size  (bp) | Ref. |
| --- | --- | --- | --- | --- | --- | --- |
| C. lectularius | | | | | | |
| ribosomal protein L18 (RPL18) | RPL18_F | GTATGACGGAGGCAGCTAGG | 93 | 56 | 137 | (Fisher et al., 2018) |
|  | RPL18_R | AACATTCGAGCAAATTCGGTA |  |  |  |  |
| DNA qPCR probe (RPL18) | Clec_ProbeVIC | [HEX]-ATGAGGACG-[ZEN]-GTGTTCTTGCCT-[TAMRA] |  | 61.5 | - | - |
| *w*Cle | | | | | | |
| 16S rRNA | INT2_F | AGTCATCATGGCCTTTATGGA | 93 | 60 | 136 | (Sakamoto and Rasgon, 2006) |
|  | INT2_R | TCATGTACTCGAGTTGCAGAGT |  |  |  |  |
| DNA qPCR probe (16S) | *w*Cle_ProbeFAM | [6-FAM]-TGGTGTCTA-[ZEN]-CAATGGGCT-[TAMRA] |  | 61.5 | - | - |
| DNA FISH probe (16S) | TsWol944R | [TAMRA]-AACCGACCCTATCCCTTCG |  | 54 | - | (Thongprem et al., 2020) |
| BEV-like | | | | | | |
| 16S rRNA | 16S_BEV-like_110F | ATACCGCATGTCGCAAGA |  | 57 | 420 | (Hosokawa et al., 2010) |
|  | 16S_BEV-like_209R | GTGAGCCATTACCCCACCTACT |  |  |  |  |
| Torix Rickettsia | | | | | | |
| gltA | RiGltA405_F | GATCATCCTATGGCA |  | 54 | 786 | (Pilgrim et al., 2017) |
|  | RiGltA1193_R | TCTTTCCATTGCCCC |  |  |  |  |

**Table S2.** Statistics for global effects of factors and their interactions on the relative quantity of *w*Cle (log) in nymphs, using the lmer model: log_10_(RQ) ~ Age_N + Feeding_State + Age_N:Feeding_State + (1|Cohort)).

| **Formula: log(RQ) ~ Age_N + Feeding_State + Age_N:Feeding_State + (1 \| Cohort)** | | | | | | |
| --- | --- | --- | --- | --- | --- | --- |
| **Linear mixed model fit by REML (lme4) and t-tests by Satterthwaite's method (lmerTest) (cells in blue)** | | | | | | |
| Data: Nymphs (instars 1-5); References: Age_N: 1^st^ instar; Feeding_State: UF | | | | | | |
| **Scaled residuals:** |  |  |  |  |  |  |
| Min | 1Q | Median | 3Q | Max |  |  |
| -2.58035 | -0.66296 | -0.02132 | 0.61371 | 2.93410 |  |  |
| **Random effects:** | | | | | | |
| Groups | Name | Variance | Std.Dev. |  |  |  |
| Cohort | (Intercept) | 0.2825 | 0.5315 |  |  |  |
|  | Residual | 0.3986 | 0.6314 |  |  |  |
| Number of obs: 153 | Groups : Cohort 6 |  |  |  |  |  |
| **Fixed effects:** | | | | | | |
|  | Estimate | Std.Error | df | t-value | Pr(>\|t\|) |  |
| (Intercept) | -0.27948 | 0.26811 | 10.34654 | -1.042 | 0.32096 |  |
| Age_N | 0.67926 | 0.05744 | 142.44194 | 11.825 | < 2e-16 | *** |
| Feeding_State1DPF | 0.29658 | 0.22499 | 142.13409 | 1.318 | 0.18956 |  |
| Feeding_State5DPF | 0.73394 | 0.22842 | 142.04595 | 3.213 | 0.00162 | ** |
| Age_N:Feeding_State1DPF | -0.09707 | 0.08343 | 142.14111 | -1.163 | 0.24659 |  |
| Age_N:Feeding_State5DPF | -0.20468 | 0.08443 | 142.05019 | -2.424 | 0.01660 | * |
| **Correlation of fixed effects:** | | | | | | |
|  | (Intr) | Age_N | F_S1DP | F_S5DP | A_N:F_S1 |  |
| Age_N | -0.494 |  |  |  |  |  |
| Feeding_State1DPF | -0.406 | 0.581 |  |  |  |  |
| Feeding_State5DPF | -0.399 | 0.569 | 0.476 |  |  |  |
| Age_N:Feeding_State1DPF | 0.336 | -0.682 | -0.835 | -0.391 |  |  |
| Age_N:Feeding_State5DPF | 0.331 | -0.670 | -0.392 | -0.837 | 0.459 |  |

**Table S3.** Statistics for global effects of factors and their interactions in adults using linear models and post-hoc analyses: **A**) lm (log(Mean_area_bact) ~ Stage); **B**) tukey_hsd (log(Mean_area_bact) ~ Stage); **C**) lm (Mean_ratio_bact ~ Stage).

| **A) lm(formula = log(Mean_area_bact) ~ Stage, data = d2)** | | | | | |
| --- | --- | --- | --- | --- | --- |
| Data: Bacteriome nymphs (instars 1-5); Reference: Stage: 1^st^ instar | | | | | |
| **Residuals:** | | | | | |
| Min | 1Q | Median | 3Q | Max |  |
| -0.64789 | -0.12178 | 0.00203 | 0.20323 | 0.54474 |  |
| **Coefficients:** | | | | | |
|  | Estimate | Std.Error | t-value | Pr(>\|t\|) |  |
| (Intercept) | 7.98205 | 0.09238 | 86.406 | <2e-16 | *** |
| Stage2 | 0.88716 | 0.15085 | 5.881 | 1.23e-06 | *** |
| Stage3 | 1.46136 | 0.13064 | 11.186 | 6.13e-13 | *** |
| Stage4 | 1.96276 | 0.14396 | 13.634 | 2.43e-15 | *** |
| Stage5 | 1.92936 | 0.15085 | 12.790 | 1.51e-14 | *** |
| Residual standard error: 0.2921 on 34 degrees of freedom | | | | | |
| Multiple R-squared: 0.8874, Adjusted R-squared: 0.8742 | | | | | |
| F-statistic: 67.01 on 4 and 34 DF, p-value: 1.204e-15 | | | | | |
| **B) Post-hoc analyses, tukey_hsd (log(Mean_area_bact) ~ Stage)** | | | | | |
| \| Stages \| group1 \| group2 \| null.value \| estimate \| conf.low \| conf.high \| p.adj \| p.adj.signif \| \| --- \| --- \| --- \| --- \| --- \| --- \| --- \| --- \| --- \| \| Stage \| 1 \| 2 \| 0 \| 0.887 \| 0.453 \| 1.32 \| 1.17e-5 \| **** \| \| Stage \| 1 \| 3 \| 0 \| 1.46 \| 1.09 \| 1.84 \| 6.04e-12 \| **** \| \| Stage \| 1 \| 4 \| 0 \| 1.96 \| 1.55 \| 2.38 \| 0 \| **** \| \| Stage \| 1 \| 5 \| 0 \| 1.93 \| 1.49 \| 2.36 \| 1.2e-13 \| **** \| \| Stage \| 2 \| 3 \| 0 \| 0.574 \| 0.140 \| 1.01 \| 4.78e-3 \| ** \| \| Stage \| 2 \| 4 \| 0 \| 1.08 \| 0.608 \| 1.54 \| 1.32e-6 \| **** \| \| Stage \| 2 \| 5 \| 0 \| 1.04 \| 0.557 \| 1.53 \| 4.82e-6 \| **** \| \| Stage \| 3 \| 4 \| 0 \| 0.501 \| 0.0869 \| 0.916 \| 1.13e-2 \| * \| \| Stage \| 3 \| 5 \| 0 \| 0.468 \| 0.0336 \| 0.902 \| 2.95e-2 \| * \| \| Stage \| 4 \| 5 \| 0 \| -0.0334 \| -0.501 \| 0.435 \| 1 \| ns \| | | | | | |
| **C) lm(formula = (Mean_ratio_area) ~ Stage, data = d2)** | | | | | |
| Data: Bacteriomes nymphs (instars 1-5); Reference: Stage: 1^st^ instar | | | | | |
| **Residuals:** | | | | | |
| Min | 1Q | Median | 3Q | Max |  |
| -2.3590 | -0.5449 | 0.1958 | 0.5395 | 2.3576 |  |
| **Coefficients:** | | | | | |
|  | Estimate | Std.Error | t-value | Pr(>\|t\|) |  |
| (Intercept) | 4.5434 | 0.3479 | 13.059 | 8.36e-15 | *** |
| Stage2 | -0.3257 | 0.5682 | -0.573 | 0.5702 |  |
| Stage3 | -0.1620 | 0.4920 | -0.329 | 0.7439 |  |
| Stage4 | -0.4711 | 0.5422 | -0.869 | 0.3910 |  |
| Stage5 | -1.1626 | 0.5682 | -2.046 | 0.0485 | * |
| Residual standard error: 1.1 on 34 degrees of freedom | | | | | |
| Multiple R-squared: 0.1206, Adjusted R-squared: 0.01717 | | | | | |
| F-statistic: 1.166 on 4 and 34 DF, p-value: 0.39 | | | | | |

**Table S4.** Statistics for global effects of factors and their interactions on the relative quantity of *w*Cle (log) during the transition between 5^th^ instar and adults, using the lmer model: lmer (log(RQ) ~ Sex + Age + Sex:Age + Sex:Age + (1|Cohort)).

| **Formula: log(RQ) ~ Sex + Age + Sex:Age + (1 \| Cohort)** | | | | | | |
| --- | --- | --- | --- | --- | --- | --- |
| **Linear mixed model fit by REML (lme4) and t-tests by Satterthwaite's method (lmerTest) (cells in blue)** | | | | | | |
| Data: Adults (nymphs 5^th^ instar-5DPF and adults 7DPF); References: Sex: Female; Age_A: 5^th^ instar | | | | | | |
| **Scaled residuals:** |  |  |  |  |  |  |
| Min | 1Q | Median | 3Q | Max |  |  |
| -1.8990 | -0.4008 | -0.1572 | 0.4739 | 1.5112 |  |  |
| **Random effects:** | | | | | | |
| Groups | Name | Variance | Std.Dev. |  |  |  |
| Cohort | (Intercept) | 0.2476 | 0.4976 |  |  |  |
|  | Residual | 0.1294 | 0.3597 |  |  |  |
| Number of obs: 26 | Groups : Cohort 6 |  |  |  |  |  |
| **Fixed effects:** | | | | | | |
|  | Estimate | Std.Error | df | t-value | Pr(>\|t\|) |  |
| (Intercept) | 1.9675 | 0.2606 | 9.9652 | 7.550 | 1.99e-05 | *** |
| SexMale | -0.3338 | 0.2159 | 17.1174 | -1.547 | 0.14027 |  |
| Age | 0.6329 | 0.2159 | 17.1174 | 2.932 | 0.00926 | ** |
| SexMale:Age | -0.3220 | 0.2891 | 17.0421 | -1.114 | 0.28078 |  |
| **Correlation of fixed effects:** | | | | | | |
|  | (Intr) | Age_N | SexMal |  |  |  |
| SexMale | -0.483 |  |  |  |  |  |
| Age | -0.483 | 0.603 |  |  |  |  |
| SexMale:Age | 0.361 | -0.747 | -0.747 |  |  |  |

**Table S5**. Statistics for global effects of factors and their interactions on the relative quantity of *w*Cle (log) in adults, using the lmer model: lmer (log(RQ) ~ Sex + Age_A + Feeding:Age_A + Sex:Age_A + Feeding:Sex:Age_A + (1|Cohort)).

| **Formula: log(RQ) ~ Sex + Age_A + Feeding:Age_A + Sex:Age_A + Feeding:Sex:Age_A + (1 \| Cohort)** | | | | | | |
| --- | --- | --- | --- | --- | --- | --- |
| **Linear mixed model fit by REML (lme4) and t-tests by Satterthwaite's method (lmerTest) (cells in blue)** | | | | | | |
| Data: Adults (Day 1-28); References: Sex: Female; Age_A: 1^st^ day; Feeding: Yes | | | | | | |
| **Scaled residuals:** |  |  |  |  |  |  |
| Min | 1Q | Median | 3Q | Max |  |  |
| -2.7011 | -0.4605 | 0.1798 | 0.6994 | 2.4409 |  |  |
| **Random effects:** | | | | | | |
| Groups | Name | Variance | Std.Dev. |  |  |  |
| Cohort | (Intercept) | 0.1467 | 0.3830 |  |  |  |
|  | Residual | 0.2808 | 0.5299 |  |  |  |
| Number of obs: 132 | Groups : Cohort 6 |  |  |  |  |  |
| **Fixed effects:** | | | | | | |
|  | Estimate | Std.Error | df | t-value | Pr(>\|t\|) |  |
| (Intercept) | 2.62166 | 0.19166 | 9.29534 | 13.679 | 1.81e-07 | *** |
| SexMale | -0.61698 | 0.15975 | 120.97851 | -3.862 | 0.000182 | *** |
| Age_A | -0.10710 | 0.05355 | 121.07329 | -2.000 | 0.047736 | * |
| Age_A:FeedingNo | -0.08873 | 0.05446 | 120.95556 | -1.629 | 0.105863 |  |
| SexMale:Age_A | 0.26707 | 0.07647 | 120.96393 | 3.492 | 0.000669 | *** |
| SexMale:Age_A:FeedingNo | -0.27557 | 0.07704 | 120.96744 | -3.577 | 0.000501 | *** |
| **Correlation of fixed effects:** | | | | | | |
|  | (Intr) | Age_N | F_S1DP | F_S5DP | A_N:F_S1 |  |
| SexMale | -0.400 |  |  |  |  |  |
| Age_A | -0.403 | 0.483 |  |  |  |  |
| Age_A:FeedingNo | -0.002 | 0.001 | -0.496 |  |  |  |
| SexMale:Age_A | 0.284 | -0.711 | -0.696 | 0.347 |  |  |
| SexMale:Age_A:FeedingNo | 0.002 | -0.002 | 0.350 | -0.707 | -0.490 |  |
